# Building the Border: Development of the Chordate Neural Plate Border Region and Its Derivatives

**DOI:** 10.3389/fphys.2020.608880

**Published:** 2020-12-07

**Authors:** Ankita Thawani, Andrew K. Groves

**Affiliations:** ^1^Department of Neuroscience, Baylor College of Medicine, Houston, TX, United States; ^2^Department of Molecular and Human Genetics, Baylor College of Medicine, Houston, TX, United States

**Keywords:** neural crest, placodes, CNS - central nervous system, PNS, signaling / signaling pathways, transcription factor, embryo, development

## Abstract

The paired cranial sensory organs and peripheral nervous system of vertebrates arise from a thin strip of cells immediately adjacent to the developing neural plate. The neural plate border region comprises progenitors for four key populations of cells: neural plate cells, neural crest cells, the cranial placodes, and epidermis. Putative homologues of these neural plate border derivatives can be found in protochordates such as amphioxus and tunicates. In this review, we summarize key signaling pathways and transcription factors that regulate the inductive and patterning events at the neural plate border region that give rise to the neural crest and placodal lineages. Gene regulatory networks driven by signals from WNT, fibroblast growth factor (FGF), and bone morphogenetic protein (BMP) signaling primarily dictate the formation of the crest and placodal lineages. We review these studies and discuss the potential of recent advances in spatio-temporal transcriptomic and epigenomic analyses that would allow a mechanistic understanding of how these signaling pathways and their downstream transcriptional cascades regulate the formation of the neural plate border region.

## Introduction

The neural plate border is one of the most developmentally complex regions in the vertebrate embryo. During the gastrulation, the epiblast begins to display signs of patterning, with the medial portion adopting a neural identity and the lateral aspect adopting a non-neural (epidermal) identity. The neural plate border region arises between the future anterior neural plate and the future epidermis in response to a series of inductive signals. Cells that intermingle at the border of the neural plate give rise to four distinct cell lineages: (1) neural progenitors that form the anterior central nervous system (CNS), (2) neural crest cells that form the peripheral nervous system, pigment cells, and much of the bone and cartilage of the face, (3) the craniofacial placodes that form complex sensory organs such as the inner ear and the olfactory epithelium, and (4) the cranial epidermis (Grocott et al., 2012; Groves and LaBonne, 2014). The fascination with this transient embryonic region is due not only to its biological significance but also to its relevance to human disease: genetic or environmental perturbations of this region collectively contribute to an enormous range of birth defects affecting the brain, skull, face, and sensory organs.

The differentiation of the neural plate border region is remarkable for several reasons. First, the four lineages generated at the border each give rise to a large number of very different cell types. Segregation of the border region into CNS neuroepithelial stem cells, neural crest cells, placodal progenitors, and epidermal stem cells requires that the four lineages become distinct from each other, while individually retaining the potential to generate a wide variety of fates within each lineage. Second, this process of segregation is extremely rapid – the events described in this review occur over a period of about 12 h in amniotes and even more rapidly in fish and amphibians, while throughout this time, the cells are dividing and changing position with respect to each other at the border. Third, the secreted inducing signals, such as BMPs, FGFs, and WNTs, that induce border region fates act over very short distances and for very short periods of time, yet somehow manage to co-operate to quickly segregate the four lineages to preclude any subsequent conversion of progenitors from one lineage into those of another. Fourth, recent studies from non-vertebrate chordates suggest that the first step in the evolution of neural crest and cranial placodes – two vertebrate novelties that appear to have arisen independently – may already have begun as the first chordates arose, and understanding the mechanisms underlying these early events may shed light on the mechanisms of border region development in vertebrates.

As gastrulation proceeds, the commitment of epiblast to one of the four border region lineages described above requires large-scale epigenetic and transcriptional changes. At present, we know almost nothing about how the chromatin of border region progenitors is rearranged and reconfigured to render some regions of the genome accessible in each lineage while simultaneously placing other regions permanently beyond use. At one extreme, the epigenome of primitive ectoderm or epiblast could gradually be transformed into multipotential cells of progressively more restricted fates, culminating in the four border lineages. At the other extreme, large scale chromatin remodeling of epiblast cells could assign them to one of these four lineages in a very short period without passing through a more multipotential intermediate. Recent advances in the ability to profile the transcriptomic and epigenetic states of individual cells mean that answering these questions may finally be experimentally tractable.

Here, we discuss the known molecular mechanisms of neural plate border differentiation, focusing on the role of morphogenetic signaling pathways and transcriptional regulators in refining boundaries between the derivatives of the neural plate border. A number of excellent reviews of the neural plate border region and its evolutionary origins have appeared in recent years, and so in addition to summarizing these mechanisms, we will also focus on a series of unresolved questions concerning the neural plate border and possible ways to address them in future.

## Development of the Neural Plate Border and the Fates and Potentials of Border Progenitors

### The Temporal Sequence of Neural Plate Border Formation

**Figure 1:**
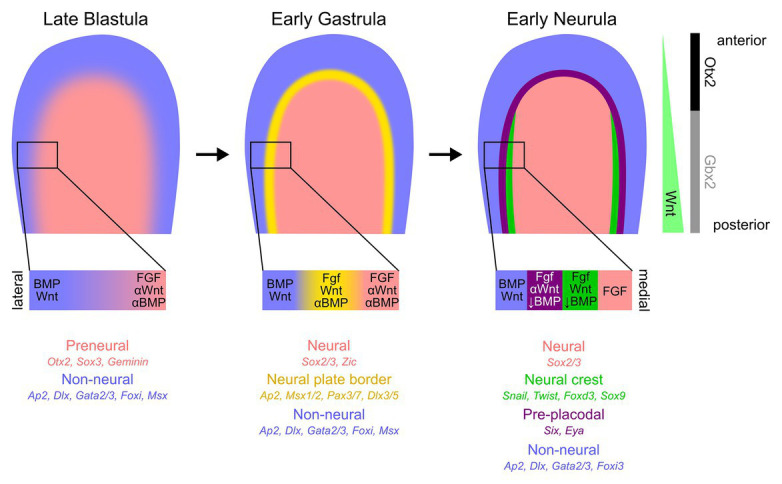
Early ectodermal patterning at the anterior epiblast. Although the ectodermal patterning varies significantly across chordates, and even within amniotes, we illustrate, here, the key stages of ectodermal patterning most faithful to amniote development. The medial epiblast begins to exhibit molecular differences compared to the surrounding tissue, with the medial region expressing pre-neural/neural (salmon) markers and lateral (blue) region with predominantly non-neural/epidermal gene expression. At the initial stages of gastrulation, the transitional zone between the neural and non-neural ectoderm, called the neural plate border (yellow), becomes more defined. By the early stages of neurulation, two distinct spatially segregated populations of cells can be detected at the border region – pre-placodal ectoderm laterally (purple) and neural crest cell progenitors medially (green). Although much remains uncertain about the roles and timing of WNT, BMP, and FGF signaling pathways and associated gene-regulatory networks during the early ectodermal patterning, a general consensus of the signaling levels and classic spatially distinct markers are indicated below the epiblast cartoons. Additionally, the asymmetric WNT signaling along the anterior-posterior axis and, subsequently, key molecular expression differences are also presented on the right-most panel.

The first evidence of division of embryonic epiblast into future neural and non-neural domains can be seen in many vertebrates prior to the onset of gastrulation. Early neural markers, such as *Otx2*, *Sox3*, *ERNI*, and *Geminin*, are expressed in dorsal ectoderm destined to give rise to the neural plate (Bally-Cuif et al., 1995; Rex et al., 1997; Kroll et al., 1998; Streit et al., 2000; Papanayotou et al., 2008). Some of these genes, sometimes referred to as “pre-neural” markers, can be induced by neural fate-inducing molecules, such as FGFs, or WNT and BMP antagonists (Streit et al., 2000; Wilson and Edlund, 2001; Albazerchi and Stern, 2007; Papanayotou et al., 2008; Rogers et al., 2011; Stern and Downs, 2012). Regions of the embryo where WNT and BMP signaling are not being actively inhibited begin to express the markers broadly considered as non-neural, such as members of the *Ap2*, *Dlx*, *Foxi*, *Gata2/3*, and *Msx* transcription factor gene families (Papalopulu and Kintner, 1993; Pera et al., 1999; Sheng and Stern, 1999; Luo et al., 2001; Knight et al., 2003; McLarren et al., 2003; Woda et al., 2003; Ohyama and Groves, 2004a; Brown et al., 2005; Matsuo-Takasaki et al., 2005; Phillips et al., 2006; Hans et al., 2007; Hoffman et al., 2007; Li and Cornell, 2007; Pieper et al., 2012; Figure 1).

As the early epiblast continues to receive signals from the organizer, additional genes considered to define neural tissue, such as *Sox2*, begin to express (Rex et al., 1997; Streit et al., 1997; Uchikawa et al., 2003). Simultaneously, expression of many non-neural genes becomes restricted to regions close to the developing neural plate (Feledy et al., 1999a; Streit, 2002; Woda et al., 2003; Khudyakov and Bronner-Fraser, 2009; Kwon et al., 2010; Pieper et al., 2012) under the influence of specific levels of BMP inhibition and FGF signaling. It is at this point that the earliest components of the neural crest gene regulatory network appear at the edge of the developing neural plate, such as *Pax* and *Zic* gene family members, *Msx1/2*, and *Ap2* (Monsoro-Burq et al., 2003; Basch et al., 2006; Hong and Saint-Jeannet, 2007; Khudyakov and Bronner-Fraser, 2009; Murdoch et al., 2010; Milet et al., 2013; Prasad et al., 2020), followed by later neural crest gene regulatory network-related members such as *Snail* and *Twist* family members, *Foxd3*, *Sox9*, and later, *Sox10* (reviewed in Prasad et al., 2012; Martik and Bronner, 2018; Hovland et al., 2020). BMP, FGF, and WNT signals derived from the future epidermis, neural plate, and mesoderm all participate in the positioning of these genes at the neural plate border (reviewed in Groves and LaBonne, 2014; Pla and Monsoro-Burq, 2018; Prasad et al., 2019; Figure 1). The source and timings of these signals varies in different vertebrate groups, but their function of inducing early neural plate border markers is generally conserved (reviewed in this Research Topic by York et al., 2020).

Shortly after the first evidence of neural crest formation, a band of ectoderm slightly lateral to the domain of neural crest markers begins to express members of the Six and Eya families (Streit, 2002; Litsiou et al., 2005; Christophorou et al., 2009; Grocott et al., 2012). This region contains undifferentiated placodal progenitors and has been termed the pre-placodal region (Grocott et al., 2012; Groves and LaBonne, 2014; Patthey et al., 2014; Schlosser et al., 2014). Some genes that initially appear to be broadly expressed in non-neural ectoderm, such as *Foxi3* and *Gata3*, refine to the pre-placodal region (Streit, 2007; Khatri et al., 2014). In contrast, *Six* and *Eya* family genes appear *de novo* in the anterior neural plate border region, extending from approximately the first pair of somites to the most anterior regions of the neural plate (Mishima and Tomarev, 1998; Esteve and Bovolenta, 1999; Kobayashi et al., 2000; Pandur and Moody, 2000; McLarren et al., 2003; Bessarab et al., 2004; Brugmann et al., 2004; Ahrens and Schlosser, 2005; Litsiou et al., 2005; Ishihara et al., 2008; Figure 1). *Six* and *Eya* gene family members continue to be expressed in many placodal derivatives as they differentiate (Xu et al., 1999; Zhu et al., 2002; Zheng et al., 2003; Bessarab et al., 2004; Zhang et al., 2004; Zou et al., 2004, 2006; Purcell et al., 2005; Schlosser, 2007; Ahmed et al., 2012a,b), but genes specific to sub-populations of placodes subsequently appear in this region in response to local inducing signals – for example, *Pax2/8* genes in the otic and epibranchial placode region, *Pax3* in the ophthalmic trigeminal ganglion, and *Pax6* in the future lens and olfactory placodes (Baker et al., 1999; Groves and Bronner-Fraser, 2000; Bhattacharyya et al., 2004; Ohyama and Groves, 2004b).

### Rostro-Caudal Patterning of the Neural Plate Border Region

**Figure 2:**
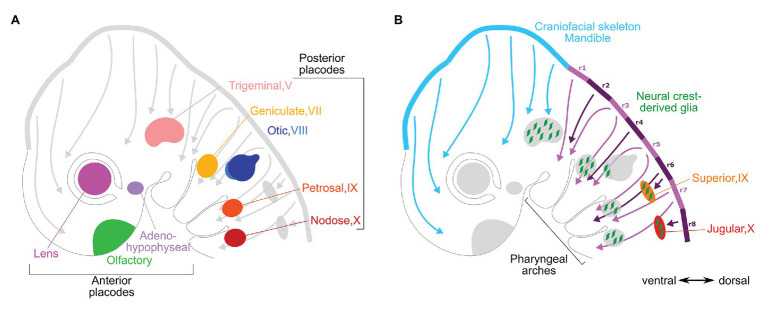
Vertebrate placodal and neural crest derivatives. Diagrams of an approximately 10-day old mouse embryo show **(A)** cranial placodal derivatives and **(B)** cranial neural crest derivatives. The arrows from dorsal to ventral sides of the embryo represent paths of the neural crest migration from the dorsal neural folds that would begin during neurulation around day 8. In both panels, the roman numerals represent the cranial nerves that the sensory ganglia are associated with: the posterior placodal-derived ganglia in **(A)** and neural crest-derived proximal ganglia in **(B)**. In panel **(B)** the rhombomeres are indicated in purple (numbered r1–r8) and midbrain and diencephalon in light blue.

Neural crest cells originating from the neural folds, delaminate during the neural tube closure, and migrate into the head and trunk to generate various skeletal and sensory structures along the anterior-posterior axis: (1) skeletal mesenchyme of the face, (2) parasympathetic ganglia and glia, (3) sympathetic ganglia and glia, (4) enteric nervous system and glia, (5) glial cells and sensory neurons of the head and dorsal root ganglia, and (6) pigment cells (Begbie, 2013). Similarly, the pre-placodal domain gives rise to multiple patches of thickened epithelium that invaginate or migrate short distances to form distinct mature derivatives from anterior to posterior end of the cranial region: (1) adenohypophysis (pituitary gland), (2) olfactory neurons, (3) lens, (4) trigeminal ganglion (cranial ganglion V), (5) inner ear, (6) epibranchial ganglia (cranial ganglia VII, IX, and X), and (7) the anterior and posterior lateral line (absent in amniotes; Figure 2; Begbie, 2013; Singh and Groves, 2016). The vertebrate cranial placodes differentiate into many cell types: sensory neurons, secondary sensory receptor cells, and secretory cells, as well as their associated supporting cells. Migrating cranial neural crest cells have a close relationship with placodal development, despite that the crest cells migrate much large distances from the neural folds compared to the placodes that thicken, invaginate, and migrate short distances. The neural crest cells populate placode-derived sensory ganglia with glial cells (see, for example, Sandell et al., 2014).

Molecular asymmetries emerge along the rostro-caudal axis during gastrulation as the ectoderm receives progressively more posteriorizing neural induction signals. *Otx2* and *Gbx2* are expressed in the anterior and posterior epiblast, respectively, and this pattern is maintained by mutual repression as the neural plate is induced and patterned, ultimately delineating the boundary between midbrain and hindbrain (Figure 1; reviewed in Wurst and Bally-Cuif, 2001). Slightly later, additional transcription factors, such as members of the Pax, Six, and Irx families, are also expressed in mutually exclusive domains along the rostral-caudal axis of the neural plate (reviewed in Gómez-Skarmeta et al., 2003). For example, Pax6 is expressed in an anterior domain of the future forebrain, whereas Pax2 is in the future midbrain, while Six1 and Irx3 sub-divide the forebrain into anterior and posterior compartments. Interestingly, some of these anterior-posterior patterning events also occur in the pre-placodal region, with Otx2 and Gbx2 being expressed in register with their domains in the neural plate, and being required for aspects of trigeminal and otic placode development, respectively (Steventon et al., 2012). Several *Pax* genes are expressed along the anterior-posterior axis in a spatially distinct pattern with *Pax6*, *Pax3*, and *Pax2/8* contributing to patterning the placodal derivatives, and also labeling them at later stages of development (Groves and Bronner-Fraser, 2000; Saint-Jeannet and Moody, 2014).

In contrast to more anterior neural patterning genes, Hox genes are not expressed in pre-placodal ectoderm and are restricted to the neural plate posterior to rhombomere 1 of the hindbrain but not to pre-placodal ectoderm. As neural crest cells migrate out of the hindbrain to populate the branchial arches, they maintain some parts of the combinatorial Hox code, but not others – for example, crest cells invading the first branchial arch express no Hox genes at all, whereas crest cells invading the second arch maintain expression of *Hoxa2* and *Hoxb2*, but not *Hoxb1* (reviewed in Parker et al., 2018). It is known that the environment into which crest cells migrate can modulate Hox gene expression (Trainor and Krumlauf, 2000a,b), but the environmental signals and transcriptional regulators that exclude Hox genes from the adjacent pre-placodal ectoderm are unknown. As progress continues to be made in understanding how signals that regulate Hox genes interact with the neural crest gene regulatory network (Parker et al., 2018), it will be of interest to understand how mechanisms that exclude Hox genes from placodal precursors arose during the evolution of neural crest and placodes in basal chordate lineages.

Why do the most anterior regions of the neural plate not generate neural crest? The olfactory placode is derived from the anterior neural fold (Couly and Le Douarin, 1985) which contains multipotent progenitors able to form epidermis, the olfactory placode and the olfactory bulb, and forebrain (Bhattacharyya and Bronner, 2013; Torres-Paz et al., 2020), but not neural crest cells. However, the anterior neural fold has transient competence to generate at least some neural crest, as grafting the chick anterior neural fold to the rostral hindbrain produces migratory neural crest. These cells also give rise to some epidermis, just as they do in their normal location (Ezin et al., 2014). The environmental signals that specifically promote placodal differentiation and repress the neural crest program in the anterior region of the neural plate border are currently unknown, although it is clear that WNT, BMP, and retinoic acid signals that affect the gross anterior-posterior pattern of the neural plate (for example, Kiecker and Niehrs, 2001) can also regulate neural crest production (Villanueva et al., 2002). For instance, knockdown of the WNT antagonist genes *Dkk* and some *Tcf/Lef* members in the anterior epiblast results in crest cell induction at the anterior neural fold (Carmona-Fontaine et al., 2007; Mašek et al., 2016).

### Unresolved Issues Concerning Lineage Segregation at the Neural Plate Border

In the previous section, we described the main gene markers whose appearance defines the neural plate, neural crest, pre-placodal region, and future epidermis. It should be emphasized, however, that the expression of many genes in the border region early stage is not uniform, with some sets of genes previously assigned as markers of a particular lineage being expressed more laterally in the border region than other markers of the same lineage. Moreover, the appearance of these markers by themselves tells us little about how and when the four neural plate border lineages segregate from each other. This is due to a paucity of fate mapping or lineage tracing data of cells at the neural plate border, and specifically an inability to correlate early marker expression with fate. For example, *Zic* and *Pax3/7* genes are considered some of the earliest markers to define the neural crest gene regulatory network (reviewed in Pla and Monsoro-Burq, 2018), yet there have been very few lineage tracing studies of these early Zic+; Pax3/7+ border progenitor cells, and the few that have been done using Cre-Lox lineage tracing suggest these cells may also contribute to placodes such as the olfactory and otic placodes (Freyer and Morrow, 2010; Murdoch et al., 2012). There is thus a clear and unmet need for genetic lineage tracing approaches to map the fate of cells expressing markers of different border region populations as well as their spatial segregation from their earliest times of expression. Moreover, only a few studies have attempted to map multiple markers for the four border lineages at early stages to test the extent to which these markers initially overlap before segregating into distinct territories (Roellig et al., 2017), although the advent of faithful multi-color mRNA visualization technologies such as RNAscope (Wang et al., 2012), Hybridization Chain Reaction (Choi et al., 2016; Roellig et al., 2017), and *in vivo* transcriptomic techniques, such as MERFISH (Chen et al., 2015), is beginning to make the simultaneous visualization of border genes more tractable.

Although these points may seem only of academic interest, they have clear implications for understanding the mechanisms of how the four border lineages segregate from each other. For example, Schlosser et al. have proposed a “binary competence” model based on grafting experiments and evolutionary considerations in which neural ectoderm exclusively is competent to form neural crest, but only non-neural ectoderm is competent to develop into placodes (Ahrens and Schlosser, 2005; Schlosser, 2008; Pieper et al., 2012). However, another school of thought of a multipotent “preborder state” proposes the induced ectoderm expresses a common set of markers during the first few hours of development and then opt a lineage specific differentiation path based on the signals received thereafter (Hintze et al., 2017; Trevers et al., 2017). The ability to label and follow the descendants of cells expressing, for example, *Zic*, *Msx*, or *FoxI* family genes, or to map the fates of *FoxD3* or *TFAP2A*-expressing cells may resolve whether the descendant cells activating expression of these genes are restricted to neural crest, placodes, or to multiple derivatives.

Another possible way of determining if a particular gene marks one of the four border lineages exclusively from early times is to perform loss-of-function studies to see if loss of a gene or gene family results in exclusive loss of a particular border lineage. In practice, however, such experiments are confounded by a number of considerations. First, some loss-of-function approaches in vertebrate embryos, such as antisense morpholinos, reduce but do not abolish gene function – thus, there are many studies reporting a *reduction* in neural crest or placode markers but not a complete loss (Woda et al., 2003; Lillevali et al., 2006; Robledo and Lufkin, 2006; Hoffman et al., 2007; Li and Cornell, 2007; Esterberg and Fritz, 2009; reviewed in Barriga et al., 2015), and there may be qualitative differences in the requirement for genes between different vertebrate groups (Barriga et al., 2015). Second, it is known that the development of a given border lineage can be regulated by its neighbors in an adjacent border derivative – for example, *Zic1* can regulate the formation of placodes in a non-cell autonomous fashion by regulating the production of retinoic acid in the neural plate (Hong and Saint-Jeannet, 2007; Jaurena et al., 2015). Despite these potential confounding factors, there are examples where loss of a single gene leads to loss of at least some derivatives specific to one of the four main border derivatives. In one such example, *Foxi3* mutant mice, deletion of the Foxi3 transcription factor leads to a complete loss of the otic placode and other posterior placodal derivatives but not anterior placodes such as the lens or olfactory epithelium (Birol et al., 2016).

### The Developmental Potential of Neural Crest Cells – Pluripotential or “Partly Pluripotential”?

Out of the four multipotential lineages that arise at the neural plate border, the neural crest is particularly striking for the diversity of derivatives it produces. This is especially true in the head, where in addition to neurons, glia, and pigment cells, neural crest gives rise to a wide range of skeletal and cartilaginous structures and specialized derivatives such as corneal endothelium more typically associated with mesoderm. These long-standing observations, together with more recent data suggesting that markers associated with neural crest specification may appear much earlier in development than previously thought (Basch et al., 2006; Betters et al., 2018; Prasad et al., 2019, 2020) have led to a renewed interest in the origin of neural crest cells and their developmental potential. Work in *Xenopus* and mice has recently shown that a number of genes previously thought to be definitive neural crest markers are actually expressed in the early amphibian blastula or mouse embryonic stem cells, and conversely, some genes typically associated with pluripotency in amphibians and mammals were expressed in the developing neural crest (Liu and Labosky, 2008; Lin et al., 2014; Buitrago-Delgado et al., 2015). Moreover, knockdown or over-expression of dominant negative versions of neural crest transcriptional effectors, such as *Snail* or *Sox5*, depleted pluripotency-associated genes in the amphibian blastula and reduced their competence to form mesoderm. Induction of a neural crest or neural plate border state by over-expression of transcription factors also extended the competence of animal cap cell descendants to form mesoderm and endoderm. Neural plate border tissue could also be induced to form endoderm in response to high concentrations of activin (Buitrago-Delgado et al., 2015). This developmental potential is regulated in part by FGF/MAPK signaling, and a transition to PI3K/Akt signaling, together with a replacement of SoxB effectors with SoxE effectors appears to drive the transition to a more developmentally restricted state (Buitrago-Delgado et al., 2018; Geary and LaBonne, 2018).

These results suggest several intriguing possibilities regarding the potential of neural crest cells and the origin of this potential. One possibility is that the neural crest is the remnants of some blastula/epiblast stem cells that were retained during gastrulation, coming to reside at the neural plate border where they could subsequently be induced to migrate and form neural crest derivatives, while still transiently possessing the competence to express mesodermal or even endodermal genes. In this model, these “set aside” cells would retain some or all aspects of the pluripotency gene regulatory network associated with the anamniote blastula or amniote epiblast. Alternatively, cells at the neural plate border could be induced to regain or recapitulate some or all aspects of the pluripotency circuit. It is certainly possible to induce the neural crest cell state *de novo* by recombining ectoderm and intermediate neural plate (Dickinson et al., 1995), treating intermediate neural plate with inducing factors (Liem et al., 1995; Garcia-Castro et al., 2002), or treating embryonic stems cells with neural- or neural crest-inducing factors (Rada-Iglesias et al., 2011; Leung et al., 2016; Gomez et al., 2019a,b; Kobayashi et al., 2020). However, technical difficulties in directly following the expression of pluripotency factors in cells as they transition from the blastula/epiblast toward neural induction and the formation of the neural plate border has made it hard to distinguish between the *persistence* of pluripotent circuits in neural crest vs. the *recapitulation* of these circuits. A recent single cell analysis of *Xenopus* embryos (Briggs et al., 2018) identified eight pluripotency genes expressed in neural crest cells (*foxd3*, *c-myc*, *id3*, *tfap2*, *ventx2*, *ets1*, *snai1*, and *oct25*) but was unable to find a unique cell cluster expressing all eight genes simultaneously, nor evidence that the eight factors were persisting from earlier precursors. Rather, this single cell analysis suggested that neural crest proceeds through a “classical” neural crest induction pathway, with neural crest arising from neuroectodermal progenitors (Briggs et al., 2018). Moreover, another recent study suggested that the amphibian *Nanog* pluripotency gene homologue, *Ventx2*, is expressed broadly in an ectodermal domain that encompasses *Pax3*- and *Zic1*-expressing crest precursors (Scerbo and Monsoro-Burq, 2020). While *Ventx2* is clearly necessary and in some cases, sufficient for expression of neural crest genes, its major role seems to be in the formation of ectomesenchymal derivatives of the neural crest (Scerbo and Monsoro-Burq, 2020), suggesting that any pluripotency or increased multipotency of crest cells by *Ventx2* may be occur secondarily to neural crest induction.

Finally, in addition to simply examining markers of pluripotency, the past 10 years have witnessed enormous progress in defining the transcriptional and epigenetic characteristics of the pluripotent state in mammals and proposing *functional* assays of varying stringency to demonstrate pluripotency *in vitro* or *in vivo* (De Los Angeles et al., 2015; The International Stem Cell Initiative, 2018). The advent of single cell technologies to analyze gene expression, chromatin accessibility, and histone modifications means that it is now feasible to compare the epigenetic and transcriptional states of stem cells as they transition toward a neural crest cell fate. This will resolve the question of which gene regulatory networks governing pluripotency are present in pre-migratory and migratory neural crest cells, and even neural crest-like peripheral glia stem cells (Adameyko et al., 2009; Dyachuk et al., 2014).

## How Inductive Signals and Transcriptional Effectors Form Distinct, Patterned Lineages at the Neural Plate Border

### Quality, Quantity, and Duration – Orchestrating Inducing Signals to Form the Neural Plate Border

Studies over the last 40 years have identified secreted signals – FGFs, WNTs, and BMPs that induce and pattern embryonic ectoderm to form the nervous system in a concentration-dependent manner. It is well-established that non-neural genes are induced in embryonic ectoderm by BMP and WNT signaling, whereas WNT and BMP antagonists from the organizer and FGF signaling promote early neural markers (Wilson and Edlund, 2001; Rogers et al., 2009) and subsequently pattern the neural plate along its anterior-posterior axis as FGF and WNT signaling are continuously modulated (reviewed in Stern, 2002; Levine and Brivanlou, 2007). Similar approaches have been used more recently to understand how the same limited set of signals are re-deployed to regulate the narrowing of the border at the edge of the neural plate and further patterning and formation of pre-migratory neural crest and the pre-placodal region (reviewed in Groves and LaBonne, 2014), and we summarize these briefly below.

Pre-placodal induction is regulated by the same signals used to induce the neural plate, although the location and timing of these signals are different from earlier stages when neural induction is initiated. FGFs, such as *Fgf8* and *Fgf4*, are expressed in cranial mesoderm underlying the neural plate border region, and removal or ectopic grafting of this mesoderm can modulate induction of pre-placodal marker genes such as members of the *Six* and *Eya* families (Ahrens and Schlosser, 2005; Litsiou et al., 2005). The role of FGFs in this cranial mesoderm was shown by gain- and loss-of-function approaches: FGF8 is sufficient to induce at least some pre-placodal genes, such as *Eya2*, in competent ectoderm, while inhibition of FGF signaling in chick and *Xenopus* can downregulate *Six* and *Eya* gene expression (Brugmann et al., 2004; Ahrens and Schlosser, 2005; Litsiou et al., 2005). WNT and BMP family members are expressed in non-neural ectoderm lateral and posterior to the pre-placodal region (*Wnt8c* and *Wnt6*: Garcia-Castro et al., 2002; Schubert et al., 2002; Kil et al., 2005; Litsiou et al., 2005; Jayasena et al., 2008; BMP4 and 7: Fainsod et al., 1994; Streit et al., 1998; Faure et al., 2002), and several antagonists of both BMPs and WNTs, such as *Cerberus* and *DAN*, are expressed in mesoderm beneath the pre-placodal region (Rodriguez Esteban et al., 1999; Ogita et al., 2001; Chapman et al., 2004; Figure 1). This suggests that downregulation or inhibition of both BMP and WNT signaling may promote pre-placodal gene expression at the neural plate border, and this was again shown by gain- and loss-of-function approaches: WNT activation reduces expression of *Six* and *Eya* genes, whereas WNT inhibition expands the expression domain of these genes (Brugmann et al., 2004; Litsiou et al., 2005). Similarly, BMPs can block expression of pre-placodal genes, whereas BMP inhibition expands the expression of pre-placodal genes (Ahrens and Schlosser, 2005; Litsiou et al., 2005; Kwon et al., 2010).

As discussed above, some genes considered to be neural crest markers are expressed in pluripotent tissue in amphibians and mammals, and some early crest markers, such as *Pax7*, are expressed in epiblast prior to formation of the definitive neural plate (Basch et al., 2006; Betters et al., 2018; Prasad et al., 2019, 2020). Nevertheless, neural crest induction has classically been considered to occur as a result of interactions between the neural plate and non-neural ectoderm (reviewed in Sauka-Spengler and Bronner-Fraser, 2008; Milet and Monsoro-Burq, 2012; Prasad et al., 2012; Begbie, 2013). FGFs, BMPs, and WNTs have been implicated in crest induction, with FGF and WNT signaling acting at early stages (Mayor et al., 1997; LaBonne and Bronner-Fraser, 1998; Villanueva et al., 2002; Monsoro-Burq et al., 2003, 2005; Stuhlmiller and Garcia-Castro, 2012), and crest induction being maintained and further promoted by WNTs from the neural folds and BMPs in the dorsal neural folds and surrounding epidermis (Garcia-Castro et al., 2002; Steventon et al., 2009; Figure 1).

### Incomplete Picture of the Interplay Between the Inductive Signaling Pathways

It has been difficult to untangle how FGF, BMP, and WNT signals are interpreted to yield very different fates at the neural plate border. Cells at the developing border region receiving these signals are within a few cell diameters of each other, and so explaining border differentiation by simple spatial gradients of these three signals cannot easily explain how such very different lineages develop in close proximity. Most of the studies described above have tended to focus on one particular neural plate border derivative and a handful of the respective lineage-specific markers without considering the effects of their experimental manipulations on other border lineages. It has also proven difficult to visualize the levels of BMP, FGF, or WNT signaling at the neural plate border in real time to observe how different cells at the border respond to these signals dynamically. Recently, Warmflash et al. have made use of simplified *in vitro* systems in which human embryonic stem cells are allowed to self-organize on micro-patterned surfaces to generate different embryonic derivatives (Warmflash et al., 2014; Deglincerti et al., 2016; reviewed in Heemskerk and Warmflash, 2016). This approach allows fine control of the level and duration of inducing signals and the patterned structures can be examined at different times to determine the timing of differentiation. This approach has recently been used to investigate patterning of the neural plate border (Britton et al., 2019). Here, the authors carefully varied the strength and duration of WNT and BMP signals to devise a protocol that used an initial phase of SMAD2/3 inhibition to simulate the differentiation of ectoderm by Nodal antagonists, followed by exposure to BMP4 and a subsequent exposure to WNT inhibitors to curtail the endogenous WNT signals arising in the micro-patterned cultures. The combined effect of this protocol produced a central zone of neural tissue (expressing Sox2, Pax6, and N-cadherin), surrounded by a ring of neural crest tissue (expressing Sox9 and Pax3), a ring of pre-placodal tissue (expressing Six1), and an outer layer of non-neural ectoderm (expressing AP2*α*, Gata3, and E-cadherin; Britton et al., 2019). These remarkable results showed the importance of precisely controlling the level and duration of BMP and WNT signaling in neural crest and placode formation but also emphasize the utility in allowing progenitor cells to achieve this differentiation through self-organization; for instance, increased WNT ligand concentration or delayed WNT inhibition in the induced ectoderm favors the neural crest fate over the pre-placodal domain. The ability to rapidly modify embryonic stem cell lines using CRISPR means that it will be possible to over-express or inactivate individual genes in this system in a temporally and spatially controlled manner, and also to use cells expressing reporters for FGF, BMP, and WNT signaling to visualize the cell-cell interactions as the neural plate border derivatives self-organize. Although undoubtedly simplified, such self-organizing *in vitro* systems are likely to generate new hypotheses for border formation that can be tested in embryos.

### Transcriptional Cross-Activation and Cross-Repression as a Mechanism for Self-Organizing Fates at the Neural Plate Border

Visualizing the formation of the neural plate border with immunostaining or *in situ* hybridization for markers of the different border derivatives shows that the boundaries between cells differentiating into different derivatives are initially very imprecise and then sharpen into clear domains (reviewed in Streit, 2007; Groves and LaBonne, 2014; Pla and Monsoro-Burq, 2018). As described above, this same process of self-organization and refinement has recently been demonstrated in micropatterned cultures of embryonic stem cells (Britton et al., 2019). Although the local cell-cell interactions that lead to this refinement are currently still poorly understood, a large body of work has suggested that transcription factor interactions within a given cell can lead to selection of one fate over another. Broadly speaking, two mechanisms underlie this transcriptional refinement: Transcription factors specific to a given lineage mutually promote each other’s transcription, while transcription factors specific to different lineages tend to mutually repress each other in the same cell (reviewed in Grocott et al., 2012; Groves and LaBonne, 2014). We summarize some of these interactions below.

Over-expression and knockdown studies in different species have demonstrated cross-repressive interactions between transcription factors expressed in early non-neural ectoderm and transcription factors expressed in the definitive neural plate. For example, over-expression of *Dlx*, *Gata*, *Msx*, *Foxi*, and *Ap2* factors repress neural markers such as *Sox2*, whereas knockdown of the same genes expand the neural plate at the expense of non-neural ectoderm (Feledy et al., 1999b; Luo et al., 2001; McLarren et al., 2003; Tribulo et al., 2003; Woda et al., 2003; Matsuo-Takasaki et al., 2005; Linker et al., 2009; Kwon et al., 2010; de Crozé et al., 2011; Pieper et al., 2012). Conversely, positive autoregulatory interactions between non-neural genes can sharpen the boundary between neural and non-neural domains (Kwon et al., 2010; Pieper et al., 2012). For example, *Ap2c*, *Foxi1*, and *Gata2* positively regulate one another’s expression in the zebrafish border region once they have been induced by BMP signaling (Kwon et al., 2010; Pieper et al., 2012; Bhat et al., 2013). As the pre-placodal region begins to differentiate, similar cross-repressive and autoregulatory interactions within this region and with adjacent non-neural ectoderm appear to refine its boundaries. For example, *Dlx* family member expression can upregulate expression of both *Six* and *Eya* pre-placodal genes and knockdown of the same *Dlx* genes can repress pre-placodal gene expression (Solomon and Fritz, 2002; McLarren et al., 2003; Kaji and Artinger, 2004; Esterberg and Fritz, 2009; Pieper et al., 2012). *Foxi3* and *Dlx5* can activate each other in chick ectoderm, and a similar positive relationship has been demonstrated between *Foxi3* and *Six1* (Khatri et al., 2014). In amphibians, Iroquois (Irx) transcription factors are expressed in the pre-placodal region immediately before *Six* and *Eya* genes and can positively regulate their expression (Gomez-Skarmeta et al., 1998; Goriely et al., 1999; Glavic et al., 2002, 2004; Khudyakov and Bronner-Fraser, 2009). Neural crest progenitors also appear to become distinct from other border derivatives by the same kinds of transcriptional dynamics. As crest cells appear at the neural plate border region, early components of the neural crest gene regulatory network, such as *Pax3* or *Pax7*, are expressed at the future neural plate border over-lapping with genes such as *Dlx5/6*, *Gata2/3*, *Foxi1/3*, *Msx1/2*, *Zic1*, *Gbx2*, and *Ap2* (Basch et al., 2006; Khudyakov and Bronner-Fraser, 2009; Murdoch et al., 2010, 2012; Grocott et al., 2012; Milet et al., 2013). Some of these genes, such as *Msx1/2*, *Zic1*, and *Foxd3*, will eventually localize with *Pax3* or *Pax7* to the neural folds where neural crest forms (Hong and Saint-Jeannet, 2005; Betancur et al., 2010) and some of these changes are driven by repression – for example, the pre-placodal gene *Six1* can repress the neural crest factors *Msx1* and *Foxd3*, whereas *Pax7* and *Msx1* repress *Six1* (Sato et al., 2010); and downregulation of *Axud1*, a WNT-responsive gene upstream of *Foxd3* neural crest program can upregulate Six/Eya expression (Simões-Costa et al., 2015).

It is important to note that the majority of these studies characterizing cross-repressive and autoregulatory interactions at the neural plate border have used over-expression of transcription factors or knockdown or dominant-negative inhibition of transcription factor function (for example, Maharana and Schlosser, 2018). There is a need for lineage tracing to directly visualize the conversion of one cell type into another as the neural plate border lineages segregate. For example, lineage tracing of Six1-expressing progenitors with Cre mice could be used to demonstrate repression of Six1 by Msx1 or Foxd3 in nascent neural crest cells – in this example, a suitably sensitive Six1-Cre line might label some pre-migratory or migratory neural crest derivatives. Similarly, the use of single cell RNA-seq technology to interrogate the border region might be able to identify intermediate cells expressing genes of two or more ectodermal derivatives as the four domains segregate from each other.

## Evolution of the Neural Plate Border in Chordates and the “New” Vertebrate Head

As animals diverged from a purely filter-feeding aquatic lifestyle to develop more complex predation behavior, the anterior head began to develop jaws (in gnathostomes) and the anterior sensory organs became more complex. This generation of a “new head” in vertebrates was a consequence of the emergence of migratory neural crest cells and invaginating placodes from the neural plate border region (Gans and Northcutt, 1983; Northcutt and Gans, 1983; reviewed in Patthey et al., 2014; Schlosser et al., 2014). It is now well-accepted that the gene regulatory networks and inductive signals that induce and pattern the vertebrate CNS are conserved to a large degree in cephalochordates (amphioxus) and urochordates (tunicates and appendicularians; reviewed in Holland, 2009). The presence of a clearly patterned anterior CNS in non-vertebrate chordates raised the controversial question of whether any cells resembling neural crest or placodal derivatives could be found in these close vertebrate relatives. It is now becoming clear from work over the past two decades that these vertebrate innovations likely began to emerge in rudimentary forms in non-vertebrate chordates, and that at least some elements of the gene regulatory networks and the secreted signals that activate them at the vertebrate neural plate border can be seen in sister chordates groups. We first describe evidence for neural crest- and placode-like elements in urochordates and cephalochordates and then summarize recent work that suggests a conserved molecular and genetic basis for their formation with vertebrates.

### Evidence for Neural Crest and Placodal Rudiments in Non-vertebrate Chordates

**Figure 3:**
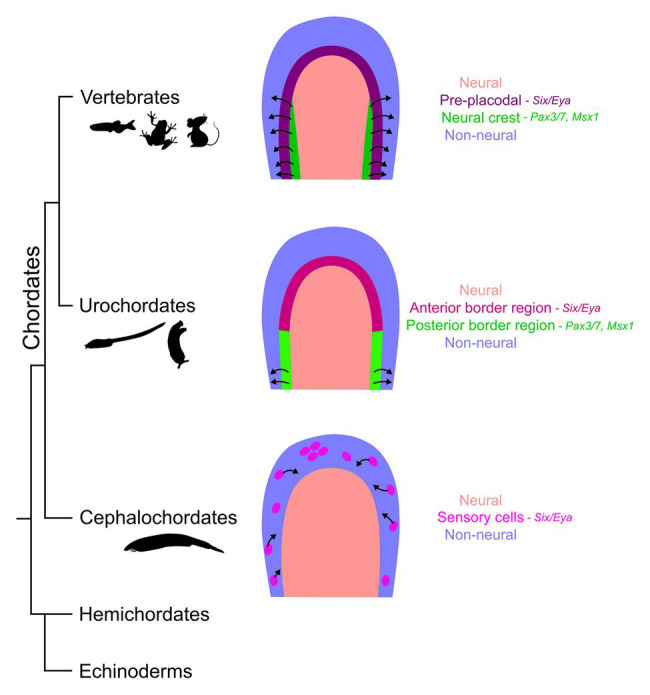
Evolution of the neural plate border in chordates. The diagrams compare the neural plate border (neural – salmon; non-neural – blue) derivatives between different taxa within the phylum Chordata. The vertebrate neural plate border gives rise to two distinct cell populations – the placodes (purple) that thicken and invaginate in the anterior embryo and the neural crest cells (green) that migrate along the entire length of the embryo except for the anterior neural fold (black arrows show migratory properties). However, this feature is an evolutionary novelty in vertebrates. The embryos from the sister clade, urochordates, have a molecularly distinct border region with several gene markers common with the vertebrates (magenta and light green); however, the crest-like migratory cell populations (light green) are relatively limited, such as the bipolar tail neurons. Cephalochordates, the phylogenetic neighbors considered less evolved to tunicate-vertebrate group, have some migratory epidermal sensory cells (pink) with similar molecular signatures to the vertebrate placodes; however, these are largely scattered individual cells that delaminate from the ectoderm much lateral to the neural/non-neural boundary.

Though urochordates and cephalochordates do not possess a complex head and numerous paired sensory organs like most vertebrates, several putative precursors of placodal and neural crest derivatives can be found (Gans and Northcutt, 1983). The presence of crest- and placode-like derivatives in amphioxus has been recently reviewed in depth by Schlosser (2017). Although amphioxus does not possess clear organized cranial sensory organs, several scattered primary and secondary sensory cells, with chemo- and/or mechanosensory roles, are spread along the anterior-posterior axis of the animal that delaminate from the non-neural ectoderm and migrate (Figure 3). Just behind the oral opening, Hatschek’s pit, a structure containing both exocrine and endocrine cells, has been proposed to be a homologue of adenohypophyseal and olfactory placodes. Some gonadotropin releasing hormone (GnRH)-expressing neurons can be observed in the CNS of amphioxus (for example, Castro et al., 2003), although the presence of GnRH reactivity in Hatschek’s pit is less certain (Castro et al., 2006). However, this organ is not derived in its entirety from the ectoderm, like the vertebrate adenohypophysis is. It is also not connected to the CNS like neurosecretory cells, thus offering key compelling arguments against this homology, as elaborated in the review by Schlosser (2017). Finally, small collections of ciliated cells, the corpuscles of de Quatrefages have been observed in anterior regions of amphioxus (Baatrup, 1982). Though hypothesized to be mechanoreceptive, the function of these cells is still uncertain (Schlosser, 2017). Although the cephalochordates do possess pigment cells and sensory neurons (Holland and Holland, 2001; Holland, 2009), migratory cells arising at the neural plate border or in the neural tube have not been observed. Rather, the peripheral sensory neurons of amphioxus arise in the ciliated epidermis, shed their cilia, and delaminate and migrate dorsally toward the neural tube before re-inserting into epidermis and sending projections in the CNS (Kaltenbach et al., 2009).

Urochordates are considered the closest extant taxa to vertebrates. The tunicate *Ciona* has become a popular non-vertebrate model system to study nervous system evolution. In its embryonic (or larval) form, *Ciona* shares many similarities with vertebrate embryonic development. Until recently, few obvious signs of neural crest-like derivatives could be observed in *Ciona* larvae. In 2012, Levine et al. reported the presence of pigmented cells in the larval CNS of *Ciona* (Abitua et al., 2012). Although these cells underwent an epithelial-mesenchymal transition, they remained in the larval neural tube. However, they could be induced to become migratory by the misexpression of the *Twist* gene that regulates migratory behavior in vertebrate neural crest (reviewed in Kuriyama and Mayor, 2008). This suggests that elements regulating two aspects of neural crest behavior – epithelial-mesenchymal transition and melanocytic differentiation – may have arisen in *Ciona* before the co-option of genes allowing those cells to become migratory. More recently, analysis of the embryonic origins of bipolar tail neurons in *Ciona* revealed that their progenitors originate in the posterior lateral neural plate ectoderm, delaminate and migrate to the middle of the larval tail, ultimately sending one process to the tip of the tail and the other to the hatchling brain (Stolfi et al., 2015; Horie et al., 2018; Figure 3). Although these migrating cells expressed some genes observed in migratory neuroblasts, such as orthologues of *Neurog* and *Isl* family members (Stolfi et al., 2015), they did not express genes typically associated with migratory neural crest. This suggests that the migratory bipolar tail neuron progenitors may represent an intermediate evolutionary phase between a neuroepithelial cell and the *bona fide* crest cells of vertebrates.

*Ciona* also has a number of cell types that can be considered homologous to anterior placodal derivatives. For example, the *Ciona* larva contains GnRH-expressing neurons that persist beyond metamorphosis (Abitua et al., 2015) and may be considered homologous to neurons derived from the olfactory or adenohypophyseal placodes of vertebrates, although evidence for expression of additional markers of these placodes in *Ciona* would provide a more solid foundation for such homology. They also contain neurons in their adhesive palps that arise from the edge of the larval neural plate (Wagner et al., 2014; Horie et al., 2018) that have been proposed to act as both chemo- and mechanosensors. In addition, cells resembling mechanosensory hair cells have been observed lining the atrial siphon of mature tunicates, where they have been proposed to help detect water flow (Manni et al., 2004; Burighel et al., 2010). The progenitors for these placode-like derivatives can be traced back to the ectoderm lateral to the neural plate (the neural plate border) of the gastrula that also express elements of the pre-placodal gene regulatory network (see below).

### Conservation of Inductive Signals and Molecular Elements of Crest and Placode Gene Regulatory Networks in Non-vertebrate Chordates

Several inductive signals that pattern the anterior epiblast and the border region that arises from the interaction of neural and non-neural ectoderm are also conserved across chordates. Similar to vertebrates, cephalochordates, and many other bilaterian taxa, employ BMP signaling to defining neural vs. non-neural identity with high BMP2/4 signaling activating epidermal fate genes, and regions of low BMP signaling, promoted by BMP antagonists (like chordin) forming a central neural ectoderm (Yu et al., 2007, 2008; Benito-Gutiérrez and Arendt, 2009; Niehrs, 2010). Similar to vertebrates, upregulation or exogenous exposure to BMP signaling in amphioxus favors the epidermal fate (Yu et al., 2008). BMP/Chordin interactions are involved in neural plate patterning in tunicates too, however, the effect of BMP over-expression is limited as early neural markers are still detected (Darras and Nishida, 2001; Lemaire et al., 2008). Protochordates also exhibit regional neural and non-neural transcription factor expression domains, neural/dorsal (SoxB and Zic), and non-neural/ventral (Dlx3/5, AP2, and Msx1) ectoderm (Sharman et al., 1999; Meulemans and Bronner-Fraser, 2004; Yu et al., 2008), molecular expression boundaries that narrow with time. In amphioxus, the lateral neural plate also expresses Pax3/7 and Snail, markers associated with neural plate border and neural crest cell development in vertebrates (Yu et al., 2008). Though, Six/Eya expression has not been observed in the amphioxus lateral neural plate or the adjacent non-neural ectoderm, Six1/2 expression has been observed in the Hatschek’s pit (possible analog to the vertebrate adenohypophysis) and epidermal sensory cell patches in late neurula stages (Kozmik et al., 2007).

Though the ascidian embryos differ from the vertebrates and amphioxus, since the cells divide and select a fate based on their lineage instead of a field of equipotent cells exposed to diffusing morphogenetic cues, the row of cells at the dorsal-most non-neural ectoderm in the *Ciona* gastrula has been shown to express *Six1/2*, *Pax3/7*, and *Msxb* (Horie et al., 2018), some of the definitive markers for the vertebrate neural plate border ectoderm (reviewed in Schlosser et al., 2014; Thiery et al., 2020). Through lineage tracing experiments and cellular fate maps of *Ciona*, we know that the *Six1* and *Foxg* cells contribute to the sensory organs like the palp adhesive organs and the oral siphon primordium (Mazet et al., 2005; Liu and Satou, 2019), whereas *Msxb* domain contributes to the bipolar tail neurons (Horie et al., 2018). *FoxG1* transcription factor labels olfactory, optic, and otic placode in vertebrates (Hebert and McConnell, 2000; Duggan et al., 2008; Ermakova et al., 2019). It has been suggested that the neural plate border of the *Ciona* embryo is compartmentalized into anterior and posterior domains with *Six1* and *Msxb* expression, comparable to vertebrate pre-placodal and neural crest progenitors, respectively. In fact, these domains can be transformed into each other in misexpression experiments (Horie et al., 2018). Additionally, another ascidian sensory patch called the atrial siphon primordium expresses *HrPax-258* (Wada et al., 1998), a homologue of the vertebrate *Pax2/5/8* gene family expressed in the otic-epibranchial placodal domain (Groves and Bronner-Fraser, 2000; Saint-Jeannet and Moody, 2014). Early neural crest cell markers like Pax3/7 are also expressed in cephalochordates and urochordates; however, many other classic neural crest cell markers like FoxD and Twist are largely absent from the ectoderm. Twist over-expression in the cephalic melanocyte lineage in *Ciona* enables those cells to migrate in a manner reminiscent of ectomesenchyme derivatives of neural crest cells (Abitua et al., 2012). In amphioxus, a defined and continuous Six/Eya-positive pan-placodal domain is absent around the neural plate compared to the other chordates. However, some Six/Eya-expressing patches can be found at later stages of development as scattered sensory cells and the Hatschek’s pit (Kozmik et al., 2007; Schlosser et al., 2014; Schlosser, 2017). Broad *AmphiMsx* expression is observed in the lateral ectoderm of the late gastrula; however, the expression becomes confined to the neural tube over time only to reappear at the presumptive location of the corpuscles of de Quatrefages at the larval stages; evidence for the lineage of these *AmphiMsx* patches is lacking (Sharman et al., 1999). Furthermore, use of an *AmphiFoxD* (homologue of vertebrate *FoxD*) enhancer to drive a GFP reporter in chick embryos shows the labeled cells localize to the neural tube, somites, and notochord but not the migrating neural crest cells, indicating that the amphioxus enhancer lacks the cis-regulatory elements for expression in migrating crest cells (Yu et al., 2008). The expression of these border region genes indicates protochordates possess the ability to segregate neural and non-neural ectoderm, with the tunicate-vertebrate clade gaining a defined border domain that gives rise to sensory cells. Despite the molecular and patterning similarities, these protochordate cells are not *bona fide* neural crest cells and placodes since they lack the capacity for invagination or long-range migration or the ability to generate various cell types of the ectomesenchyme, as seen in the vertebrate head.

Both neural crest cells and placodes arose around the same time during evolution, generate some similar cell types (sensory neurons), and a portion of these cells possess migratory properties. Furthermore, as described above, these cells in the invertebrate chordate lateral non-neural ectoderm or the neural ectoderm express transcription factors homologous to the neural crest progenitors and pre-placodal domain. This raises the question of whether basal chordates or early vertebrates possessed a hybrid neural border cell population, a common ancestral cell type to neural crest and pre-placodal progenitors (reviewed in Schlosser, 2008).

### Probing an Ancient Border With Modern Tools

The invertebrate chordates do not have placodes or neural crest cells similar to the vertebrate taxa; however, scattered cells and patches that possess comparable molecular characteristics and sometimes migratory properties of ectodermal patterning during late gastrulation/early neurulation can be used to track the origins of the gene regulatory networks that gave rise to the neural plate border of vertebrates. Comparative studies across the evolutionary spectrum can also shed light on the role of signaling pathways and transcription factors for cell type-specific development. For example, single cell RNA- and ATAC-sequencing of mammalian hair cells and the ciliated primary sensory cells of the ascidians could compare the transcriptomic and epigenomic regulation of fate specification in the two taxa.

Another unaddressed question that has been debated for several decades is whether neural crest cells and the pre-placodal domain share a common evolutionary origin. The two cell populations form adjacent to each other at the neural plate border during early epiblast development, the cells possess migratory properties, they migrate along the same “corridors” during neurulation (Steventon et al., 2014), and the two lineages share some resultant cell fates like sensory neurons. Fine signaling pathway or transcription factor misexpressing easily transforms one cell type to another as demonstrated, for example, in tunicate embryos in Horie et al. (2018) and *in vitro* ectodermal patterning by Britton et al. (2019). A detailed review by Schlosser (2008), however, argues against this, since the neural crest cells are specified and migrate from the neural plate border before the placodes, and many cell fates are exclusive to each of the lineages – only neural crest cells make bone and cartilage, while only placodes form specialized mechano- or chemoreceptive sensory cells. Also, the defining gene regulatory networks of placodes (Six/Eya) and neural crest cells (Pax3/Msx/FoxD) are quite different. For further evidence for the interplay of these gene regulatory networks, we need a comprehensive comparison of active, repressed, and accessible genome loci across the two neural plate border lineages during late gastrulation and early neurulation stages. Single cell transcriptomics studies in *Ciona* show that misexpression of anterior lateral plate border genes (like *Foxc*) in the posterior counterpart (*Pax3/7*) result in transformation to the anterior fate along with a significant number of cells with a hybrid anterior-posterior lateral plate border transcriptome (Horie et al., 2018), supporting the theory of a multipotent intermediate cell population at the border that gives rise to crest-like and placode-like cells.

The question remains as to what mechanisms localized the proto-neural plate border gene regulatory network to the edge of the neural plate during evolution. How did transcription factors that define border populations such as *Six/Eya/Pax* genes gain regulatory elements regulated by WNT/BMP/FGF signaling that allowed their expression in a domain separate from neural and non-neural ectoderm? Many such questions remain to be explored in the field of early ectodermal patterning in chordates and with the emergence of novel tools to probe gene expression and regulation at the single cell level, we can continue to piece together the mystery of neural plate border induction, specification, and lineage commitment.

## Conclusion: Toward a Multi-Omics Investigation of the Neural Plate Border Region

Many questions in the field of neural plate border development remain to be addressed. How do cells segregate from pluripotential epiblast cells to one of four lineages; each remaining multipotent but nonetheless distinct and restricted in their fates from the other lineages? What is the precise combination of WNT/BMP/FGF signaling that gives rise to each of the four ectodermal cell fates, and what is the downstream signaling cascade for each lineage? Do the neural crest progenitors and pre-placodal ectoderm arise from a common pool of cells at the border region in a hybrid stage, or do they come from the neural and non-neural ectoderm respectively? Is the chromatin conformation of the border irreversible at early developmental stages when the first neural crest cell or pre-placodal marker expression is observed, or does it remain plastic? Do neural crest cells retain some or all aspects of pluripotency? How similar are the cell types that originate in the non-neural ectoderm adjacent to the neural plate in protochordates to those in vertebrates that generate sensory patches and organs? To address these questions, we need to follow the transcriptomic and epigenomic states of cells at the neural plate border in the medial-lateral as well as anterior-posterior axes.

Embryonic cellular maps are difficult to construct due to the rapidly changing nature of embryonic tissue, unlike the atlases of adult organs. Histochemical methods can only reveal spatial expression of certain known genes and proteins in the organism, with further limitations pertaining to lack of cross-species utility of those tools. Introduction of single cell sequencing techniques in the last decade has finally made it possible to observe a more holistic picture of a developing cell and its state at a particular point in embryonic time. Single-cell level transcriptomic, epigenomic, and other sequencing techniques provide the necessary apparatuses to capture the dynamic state of the developing embryo to track lineages, observe fate specification, and study the multipotency of the differentiating cells. Several recent studies present a comprehensive atlas of different cell types of the developing mouse embryos pre-gastrulation (Mohammed et al., 2017; Cheng et al., 2019) and from gastrulation to organogenesis (Pijuan-Sala et al., 2019). Statistical clustering of single cells can be used to discover previously unidentified or non-distinct cell populations as well as observe detailed similarities and differences in the transcriptional states of known cell types. Molecular maps of cell lineage induction and specification can be gathered by profiling the tissue of interest from multiple ages simultaneously, which can further help to understand the gene regulatory networks involved. For example, Pijuan-Sala et al. (2019) show the origins of endodermal lineages in the mouse embryo during gastrulation and identify a population of early myeloid progenitors that can contribute to microglia. A similar analysis in tunicate embryos permitted virtual lineage tracing of the nervous system. This transcriptomic study revealed molecular similarities between the vertebrate telencephalon and the anterior domain of the tunicate embryo (palp sensory cells and anterior sensory vesicle) supporting the idea that protochordate ectodermal gene regulatory modules must have evolved to expand the vertebrate forebrain (Cao et al., 2019). It will be possible to compare the transcriptomic profile of bipolar tail neurons, atrial siphon primordium, oral siphon primordium, and palp organs with vertebrate transcriptomes of crest and placode derivatives to comprehensively explore the parallels between vertebrate and protochordate neural plate border ectoderm development.

As a complement to single cell RNA sequencing, multi-color imaging methods like RNAscope or *in situ* Hybridization Chain Reaction can be used to record and validate spatial expression of key genetic variations observed in cell clustering (Choi et al., 2016; Lignell et al., 2017). The neural plate border region of the chordates is only a few cells thick and techniques like Slide-seq and MERFISH may be able to give higher resolution spatial information of the cellular transcriptome during border development (Chen et al., 2015; Rodriques et al., 2019). While the former method transfers single cell thick tissue sections onto a single cell RNA sequencing grid (Rodriques et al., 2019), the latter method probes the same tissue sections for thousands of RNA transcripts by using a robust multichannel ISH technique (Chen et al., 2015); both methods provide a spatial context to the transcriptomic profiles. Depending on the cellular resolution of these techniques, they may be useful for parsing out the cellular identities at the neural plate border region where the four ectodermal lineages are intermingled over just a few cell diameters. For instance, although WNT, BMP, and FGF signaling are important for neural crest progenitors and pre-placodal domain patterning, far less is known of the fine-tuned signaling levels and subsequent cascade of downstream molecules that specify these cell fates. Single cell RNA sequencing techniques can elaborate upon the levels of signaling and expression of respective downstream effectors to delve deeper into the signaling pathway interactions at the neural plate border. Advances in transgenic animal models, CRISPR technology, and high-resolution live imaging of fluorescent reporter of cell signaling can help us visualize signaling spatiotemporally. For example, we can evaluate whether an intermediate cell fate that can give rise to both neural crest cells and placodes exists in the developing epiblast, thereby testing the “binary competence model” of ectodermal patterning (Patthey et al., 2014; Schlosser et al., 2014).

Ideally, we would want to interrogate the expression levels and cell state of every cell in an embryo at a chosen state of development and be able to track each individual cell through time and space. However, sequencing technologies are often not a comprehensive picture of the cell states with anywhere between 16 and 62% coverage of the cells at the early embryonic development stages observed with each individual cell identified with only a couple of thousand transcripts (Tam and Ho, 2020). Although it is enough to cluster the single cells identified, the studies may give an incomplete picture of the transitional states due to insufficient sequencing depth. With the realistic limitations of the technology, integrating databases across published studies can help us trace the lineage of cell clusters along the developmental timeline (Tam and Ho, 2020). Alternatively, higher sensitivity but lower throughput techniques, such as MATQ-seq and SMARTer-seq, can detect a higher number of genes per cell and are useful for a deeper transcriptomic analysis of single cells (Sheng et al., 2017; Verboom et al., 2019). For example, a recent study uses SMART-seq2 to understand the fate programs of neural crest cells with over 7,000 genes detected per cell for a finer understanding of the transcriptomic decisions made by pre-migratory/migratory crest cells as they proceed toward sensory, glial, or mesenchymal fates (Soldatov et al., 2019).

Several key transcription factors like *FoxD3*, *Foxi3*, *Pax3/7*, and *Zic* have been implicated in broader patterning of the ectoderm around the neural plate border (reviewed in Pla and Monsoro-Burq, 2018). Techniques like ChIP-seq and CUT&RUN can identify the genomic loci bound by a transcription factor of interest, or which genomic loci are “primed” by particular histone modifications (Skene and Henikoff, 2017; Kaya-Okur et al., 2019). Comparison of the transcriptomic and epigenomic data from the neural plate, neural crest, and pre-placodal domain cells can identify “active” transcription loci (SCENIC; Aibar et al., 2017). In addition to the transcriptomic status of the cells at the developing neural plate border region, it remains to be addressed when the cells are fully committed to a lineage. Epigenomic sequencing analysis, such as ATAC-seq, can identify accessible genomic loci available for transcriptional activity. Single cell ATAC-seq is now feasible, and it is now possible to combine scRNA-seq and ATAC-seq in a single cell (for example, Cao et al., 2018; Rosenberg et al., 2018; Reyes et al., 2019). Using this data, we can identify relevant enhancers for lineage specific transcription factors and evaluate plasticity of the cells, whether trans-differentiation is feasible from one ectodermal path to another. For example, combining single cell RNA-seq, ATAC-seq, and ChIP-seq data, Lukoseviciute et al. (2018) identified a bimodal function for *FoxD3*, a key transcription factor that is important for neural crest specification and differentiation. Their data show that FoxD3 binds to cis-regulatory elements for neural crest specifier genes as an activator, and at later stages, represses mesenchymal and migratory programs to prevent premature differentiation (Lukoseviciute et al., 2018). Yet another transcription factor, AP2 (or TFAP2) has been shown to play a dual role in activating neural crest induction genes (*Pax*, *Zic*, and *Msx*) and, at a later stage of development, neural crest specification genes (*FoxD* and *Sox10*). Combining multiple sequencing efforts to collate single cell RNA-seq, ATAC-seq, and AP2 CUT&RUN data shows that this transcription factor performs these two distinct roles based on its dimerization partner (Rothstein and Simoes-Costa, 2020). The ability to interrogate the gene expression, histones, nucleosome availability, and more recently, genome occupancy is finally shedding light on the role of such key transcription factors and signaling cascades for ectodermal patterning to generate and specify distinct lineages.

## Author Contributions

AT and AG wrote and edited the manuscript together. Both the authors contributed to the article and approved the submitted version.

### Conflict of Interest

The authors declare that the research was conducted in the absence of any commercial or financial relationships that could be construed as a potential conflict of interest.
